# Mature teratoma of the anterior mediastinum revealed by supravalvular pulmonary stenosis: a case report

**DOI:** 10.11604/pamj.2022.43.109.32333

**Published:** 2022-10-28

**Authors:** Hafedh Daly, Amira Horchani

**Affiliations:** 1 Faculty of Medicine, Cardiovascular Surgery Department, Monastir, Tunisia,; 2 Faculty of Medicine, Pharmacy Department, Monastir, Tunisia

**Keywords:** Teratoma, mature, mediastinum, supravalvular pulmonary stenosis, case report

## Abstract

The mature teratoma of the mediastinum is a benign tumour made up of several adult-type tissue components which result from the abnormal development of two or three embryonic layers (ectoderm, endoderm or mesoderm). We report the case of a 31-year-old patient, admitted for management of a mediastinal tumour, revealed by a pulmonary supravalvular stenosis, symptomatic of exertional dyspnea and mid-thoracic pain. The clinical examination objectified a respiratory rate at 18 c/min and an oxygen saturation at 96% in ambient air. The chest X-ray showed a mediastinal enlargement with a left paracardiac opacity, while the echocardiac showed a supravalvular pulmonary stenosis, hence the realization of a thoracic computed tomography (CT), which objectified a voluminous anterior mediastinal cystic formation and superior lateralized on the left, with an effect of compression on the trunk of the pulmonary artery. The median sternotomy allowed the complete exeresis of this tumor and the postoperative course was simple. The mature teratoma of the anterior mediastinum should be suspected in view of these clinical and radiological signs in a young patient. Surgery with a complete resection remains the treatment of choice.

## Introduction

Teratomas are a part of the non-seminomatous tumors and correspond to formations derived from one or more of the three embryonic sheets (ectoblast, entoblast and mesoblast) [1]. They can be cystic or solid, mature or immature, and have benign or malignant behavior [2]. Benign forms correspond to mature teratomas which account for 50-70% of mediastinal germ cell tumours, 10% of mediastinal tumours and 80-88% of mediastinal teratomas [3]. They are composed exclusively of well-differentiated adult-type tissues [4]. They usually sit at the anterior mediastinum and are often asymptomatic, which underestimates their true proportion [1]. We report a case of mature teratoma manifesting essentially by signs of supravalvular pulmonary stenosis.

## Patient and observation

**Patient information:** this is a 31-year-old patient with no known pathological history, admitted for the management of a mediastinal tumor.

**Clinical findings:** the clinical examination objectified an asthenic patient, a weight of 63 kg with notion of unquantified weight loss, blood pressure of 125/70 mm Hg, a heart rate of 85 cycles per minute, a normal cardiac auscultation, a respiratory rate of 18 cycles per minute and an oxygen saturation of 96% in ambient air.

**Timeline of current episode:** the patient reported exertional dyspnea, evolving for one year, then the onset of intermittent mid-thoracic pain for 3 months.

**Figure 1:**
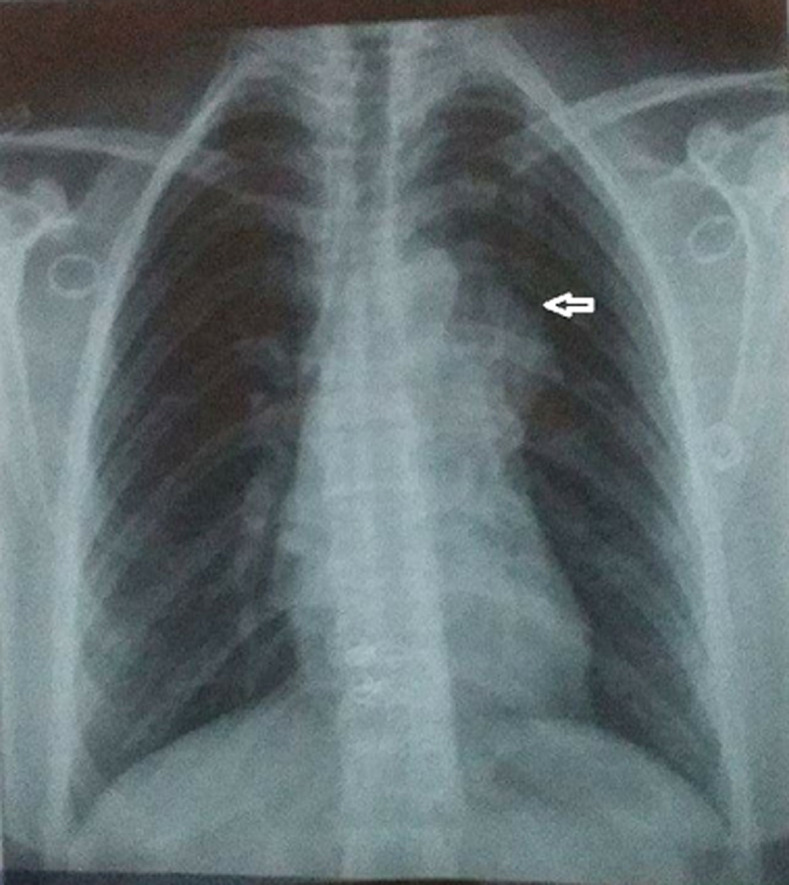
preoperative chest X-ray; left paracardiac opacity (arrow)

**Figure 2:**
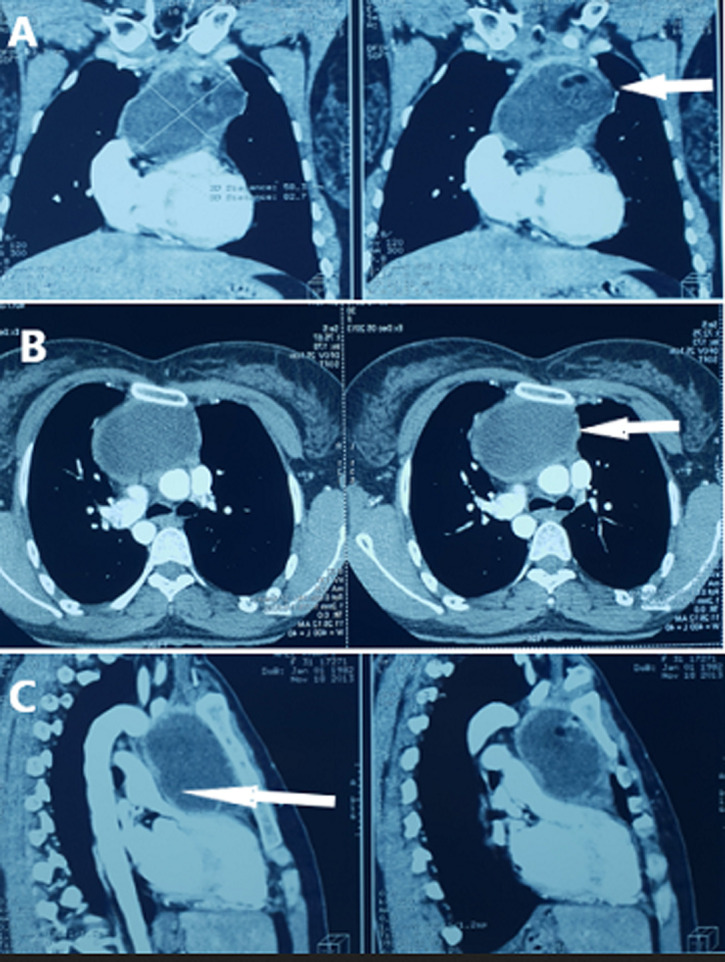
frontal computed tomography: (A) mature teratoma of the superior mediastinum; axial computed tomography; (B) mature teratoma of the anterior mediastinum; sagittal computed tomography; (C) mature teratoma that compresses the trunk of the pulmonary artery (arrow)

**Diagnostic assessment:** the chest X-ray showed mediastinal enlargement with left para-cardiac opacity (Figure 1). The cardiac ultrasound was done in front of this dyspnea, showed a preserved left ventricular function, the absence of valvulopathies and the presence of a supravavular pulmonary stenosis. The chest computed tomography scan (CT) objectified a voluminous anterior and upper mediastinal cystic formation lateralized to the left of 9x7.2x6 cm with a compression effect on the trunk of the pulmonary artery (Figure 2).

**Figure 3:**
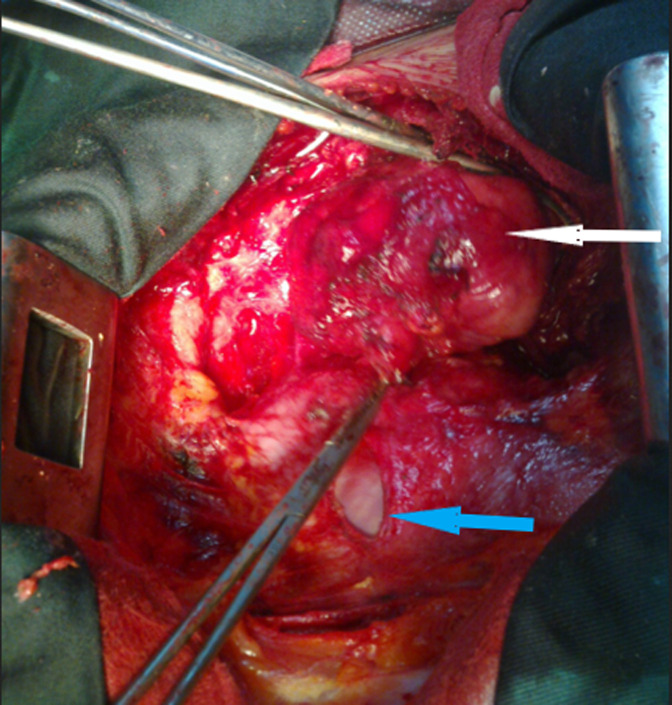
intraoperative view: mature teratoma of the anterior and superior mediastinum (white arrow); the heart (blue arrow)

**Figure 4:**
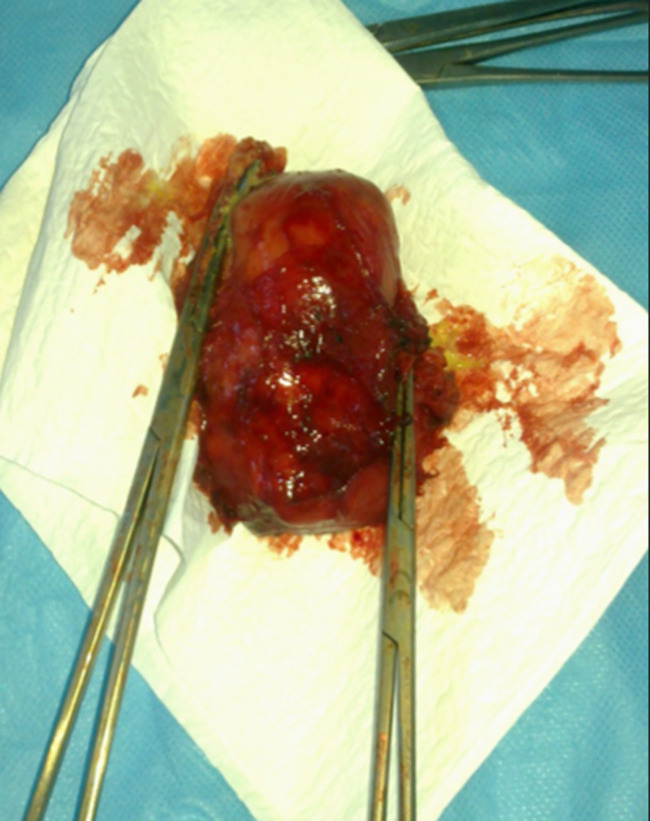
complete excision of the mature teratoma

**Therapeutic intervention:** the patient was operated by median sternotomy. The exploration found a mediastinal mass of 9 cm of major axis, related to the lateral face of the aorta, with the trunk of the pulmonary artery and very adherent to the pericardium (Figure 3). The dissection was very difficult due to the intimate adhesions with the surrounding structures. The meticulous dissection allowed the complete excision of this lesion (Figure 4). The pathological study confirmed that it was a mature mediastinal cystic teratoma containing sebaceous gland-rich skin tissue, smooth adipose and muscle tissue.

**Figure 5:**
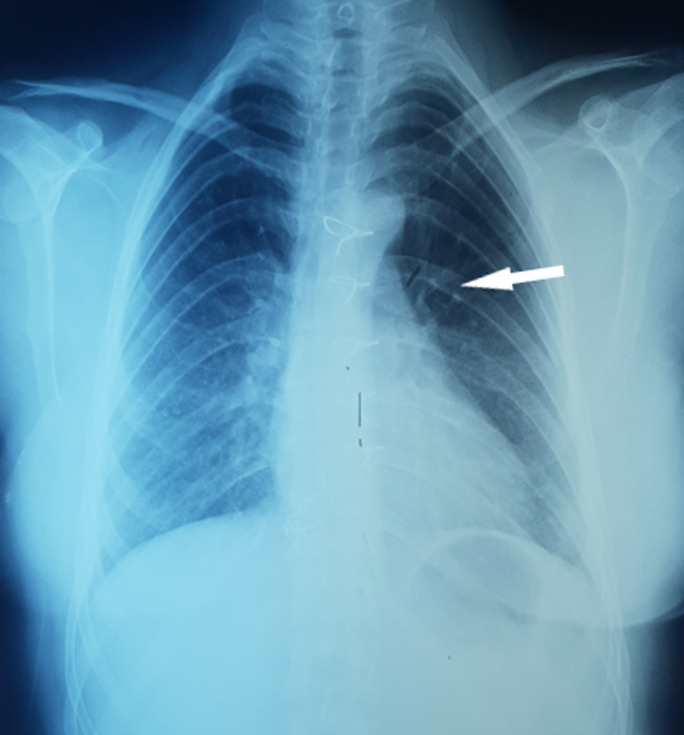
postoperative chest X-ray; disappearance of left paracardiac opacity (arrow)

**Follow-up and outcomes:** the decline is currently 6 years. The patient is asymptomatic with 100% saturation in ambient air without any dyspnea or chest pain. The chest X-ray did not objectify any detectable abnormality (Figure 5).

**Patient perspective:** during her hospitalization and after treatment, the patient was satisfied with the care she received and optimistic about the progress of her condition.

**Informed consent:** it was obtained from the patient.

## Discussion

The mediastinum represent the second favorite site of teratomas after the gonads. They concern, in most cases, the anterior mediastinum and often occur in the young subject, between 15 and 30 years, of both sexes with a female predominance [5]. No predisposing factors or associated conditions have been recognized in subjects developing these tumors, even though immature teratomas occur almost exclusively in males [6]. In adults, these teratomas are often asymptomatic [7]. It is on the occasion of a complication that the teratoma will be the cause of functional signs due to tumor growth with neighborhood compression, tumor rupture, infection, pleuropulmonary complications or malignant degeneration [8]. In our case, the dyspnea is due to the supravalvular pulmonary stenosis (extrinsic compression of the trunk of the pulmonary artery by the tumor). The imaging retains its place in the positive diagnosis of mediastinal teratomas, which often remain asymptomatic. The teratoma usually appears on the chest X-ray as an anterior, rounded, ovoid or polylobed mediastinal mass well circumscribed and often lateralized to one of the hemithorax. The computed tomography accurately assesses the density of all included tissues such as soft tissues, fluids, fat, calcifications and teeth. Sometimes radiological diagnosis can be difficult in complicated teratomas [9]. The magnetic resonance imaging (MRI) is a very valuable tool for detecting anatomical relationships with the mediastinum and hilar structures such as vessels and airways [9,10]. The originality of this case is based on its size (9 x 7.2 x 6 cm) and on the compressive character of adjacent organs related to mechanical effects, including the aorta and trunk of the pulmonary artery.

The complete and careful surgical resection of mediastinal teratomas remains the only possible therapeutic approach in order to avoid complications and local recurrences [10,11]. This complete excision is sometimes made difficult by the large size of these tumors and the intimate adhesions with neighboring structures, especially with the pericardium, lung, large vessels, thymus, chest wall, hilar structures or diaphragm [12]. In our case, despite the adhesion of the teratoma with the trunk of the pulmonary artery, we were able to perform complete resection without recourse to extracorporeal circulation. The prognosis of mature teratomas of the mediastinum is excellent after complete resection. Recurrences are exceptional mainly related to incomplete resection and can occur in a benign or rarely malignant tumor form, hence the interest of regular follow-up based on clinical examination, radiological assessment, serum assay of α-fetoprotein and β-HCG [6,13].

## Conclusion

Giant mature teratomas of the mediastinum remain rare tumours, they cause mechanical compression of the adjacent structures, causing clinical manifestations. They should be suspected in front of a parasternal opacity on the chest X-ray in a young patient. The complete and careful resection is the treatment of choice to avoid complications and local recurrences, with a prognosis that remains favorable.
